# A Comprehensive Study on Pain Assessment from Multimodal Sensor Data

**DOI:** 10.3390/s23249675

**Published:** 2023-12-07

**Authors:** Manuel Benavent-Lledo, David Mulero-Pérez, David Ortiz-Perez, Javier Rodriguez-Juan, Adrian Berenguer-Agullo, Alexandra Psarrou, Jose Garcia-Rodriguez

**Affiliations:** 1 Department of Computer Technology, University of Alicante, 03080 Alicante, Spain; mbenavent@dtic.ua.es (M.B.-L.); dmulero@dtic.ua.es (D.M.-P.); dortiz@dtic.ua.es (D.O.-P.); jrodriguez@dtic.ua.es (J.R.-J.); aberenguer@dtic.ua.es (A.B.-A.); 2 School of Computer Science and Engineering, University of Westminster, 115 New Cavendish Street, London W1W 6UW, UK; psarroa@westminster.ac.uk

**Keywords:** pain assessment, computer vision, deep learning, sensor data, signal processing, pattern recognition

## Abstract

Pain assessment is a critical aspect of healthcare, influencing timely interventions and patient well-being. Traditional pain evaluation methods often rely on subjective patient reports, leading to inaccuracies and disparities in treatment, especially for patients who present difficulties to communicate due to cognitive impairments. Our contributions are three-fold. Firstly, we analyze the correlations of the data extracted from biomedical sensors. Then, we use state-of-the-art computer vision techniques to analyze videos focusing on the facial expressions of the patients, both per-frame and using the temporal context. We compare them and provide a baseline for pain assessment methods using two popular benchmarks: UNBC-McMaster Shoulder Pain Expression Archive Database and BioVid Heat Pain Database. We achieved an accuracy of over 96% and over 94% for the F1 Score, recall and precision metrics in pain estimation using single frames with the UNBC-McMaster dataset, employing state-of-the-art computer vision techniques such as Transformer-based architectures for vision tasks. In addition, from the conclusions drawn from the study, future lines of work in this area are discussed.

## 1. Introduction

Pain is an unpleasant sensation that individuals may encounter during their lifetime. It is related to the nervous system and operates as an alert, signaling potential injury or damage to the body. Consequently, seeking medical assistance is a common outcome of pain. There are different types of pain based on its properties and duration (acute or chronic, nociceptive or neurophatic, etc.), each producing different sensations [1,2].

Pain plays a pivotal role in human existence, serving as a crucial indicator of potential health issues. In addition, if persistent, it may lead to disruptions in daily activities, causing frustration, depression, or sleep disturbances as remarked in [3,4,5,6]. However, pain remains a subjective experience, making unbiased evaluations difficult since it cannot be quantified like temperature, volume, pressure, or other objective parameters. Nevertheless, efforts have been made to create rating systems that enable healthcare providers to evaluate and treat patients using quantitative measures. In recent years, various scales and surveys have been put forth for this very purpose. These scales encompass the Official Belgian Disability Scale, the AACS scale (Argentinean Association of Insurance Companies), the Numerical Rating Scale (NRS), and the Verbal Rating Scale (VRS), among others. Within these widely employed scales, several significant challenges emerge. Chief among them is the issue of subjectivity, which stems from the inherent nature of pain as a subjective experience. In many instances, patients may convey information that is not entirely accurate. For instance, in the NRS classification, a considerable degree of subjectivity persists, as it relies on the patient’s self-assessment to assign a pain intensity rating within a range of 0 to 10 as depicted in Equation (1). This introduces a considerable degree of subjectivity. Moreover, individuals belonging to specific groups, particularly those with cognitive impairments, may encounter difficulties in effectively communicating their pain, even when simplified scales are applied:

(1) $$None=\left\{0\right\}\;\;Mild=\left\{1,2,3\right\}\;\;Moderate=\left\{4,5,6\right\}\;\;Severe=\left\{7,8,9,10\right\}$$

Given the inherent subjectivity of pain experiences, alternative methods have been developed to leverage observable cues, such as facial expressions, body movements, or vocalizations exhibited by individuals. One such scale, created with this purpose in mind, is the Pain Assessment in Impaired Cognition (PAIC) scale [7]. PAIC, centered on the observation of various pain indicators, is designed to offer an objective assessment of pain-related signs that can be discerned in cases where patients are unable to verbally communicate their discomfort. These indicators are evaluated from 0 (none) to 3 (high), depending on the degree of pain expressed, and encompass the following:

- Facial expressions: People often convey their emotional state, as well as their emotional well-being, through facial expressions. Analyzing these facial expressions is a method for assessing whether the subject is experiencing any form of discomfort or distress. Such expressions include actions such as frowning, raising the upper lip, or opening the mouth.
- Body movements: People may move the body in different ways in reaction to pain. Some common activities are rubbing, showing restlessness or protecting the affected area.
- Vocalization: Despite not seeking verbal communication, some vocal expressions like “ouch” indicate pain or express discomfort.

Another noteworthy scale is the Prkachin and Solomon Pain Intensity Scale (PSPI) [8], based on the FACS (Facial Action Coding System) [9]. PSPI endeavors to quantify an individual’s pain levels by examining their facial expressions, specifically focusing on the facial movements most pertinent to pain-related situations. A calculation is then derived based on these observations [8,10]. Equation (2) depicts the formula used to derive the values on the PSPI scale. Specifically, AU4 corresponds to frowning, AU6 and AU7 pertain to orbital tension, AU9 and AU10 relate to lip elevation, and AU43 is associated with eye closure:

(2) Pain=AU4+(AU6||AU7)+(AU9||AU10)+AU43

Hence, it can be deduced that the PSPI scale holds notable significance in the context of pain measurement via facial expressions. It boasts several favorable attributes, including the capacity for objective pain assessment and a scoring range from 0 to 16, providing enhanced precision in pain evaluation. As a result, it is chosen as the preferred method within the methodologies developed and the datasets selected for this task’s advancement.

In this work, we present a comprehensive study over two popular datasets: UNBC-McMaster [10] and BioVid [11]. These datasets have been widely recognized for their significance in pain assessment research. By conducting our study on both of these datasets, we aim to provide a robust and comparative analysis of different pain assessment methodologies. Furthermore, leveraging these two benchmark datasets enables us to establish a baseline for pain assessment techniques, which can serve as a reference point for future research endeavors in this field. This benchmarking process is vital in objectively evaluating the performance of novel methodologies and understanding their relative strengths and limitations. In the subsequent sections, we delve into the specifics of our methodology, detailing the analysis of data extracted from biomedical sensors, as well as the application of state-of-the-art computer vision techniques for facial expression analysis. We then present the comparative results of these approaches, providing valuable insights into their respective contributions to pain assessment. The contributions of this work are summarized as follows:

- Thorough examination of correlations extracted from diverse biomedical sensors within the BioVid dataset to understand interdependencies.
- Precise pain level assessment, utilizing state-of-the-art computer vision algorithms on static facial expressions of participants.
- Enhancement of analysis accuracy by integrating temporal context through video data, transitioning from static to dynamic analysis.
- Establishment of a foundational baseline for future research in pain assessment while advocating for the use of multimodal data.

The remaining of this paper is organized as follows. Section 2 provides an overview of relevant literature on pain assessment. Section 3 provides an extensive analysis on the BioVid dataset. In Section 4 and Section 5, the UNBC-McMaster and BioVid datasets are used for image and video classification of pain assessment, respectively. The limitations, strengths and future directions for pain assessment are discussed in Section 6. Finally, conclusions from this work are summarized in Section 7.

## 2. Related Works

Pain, due to its subjective nature, presents a considerable challenge when it comes to evaluation. Existing methods for assessing pain predominantly hinge on an individual’s ability to identify and communicate their experienced pain event. Yet, the perception and articulation of pain are influenced by a multitude of factors encompassing personality attributes, as well as physical and psychological well-being. Consequently, various strategies have been suggested for the automated detection of pain intensity, leveraging quantifiable physiological and audiovisual indicators. Building upon the aforementioned fact, the subsequent sections present the most relevant databases in pain assessment, followed by an exploration of the methodologies developed for automatic pain assessment.

### 2.1. Datasets

Among the initial and highly notable databases dedicated to pain made available to the research community is the UNBC-McMaster Shoulder Pain Expression Archive Database [10]. The database comprises 129 individuals experiencing shoulder pain, engaging in prescribed motion exercises using both their affected and unaffected limbs. Throughout these exercises, video footage was captured to document the participants’ natural facial expressions. The authors provided annotations for each video frame using both the Facial Action Unit System (FACS) [9] and the Prkachin and Solomon Pain Intensity (PSPI) [8] metrics. Besides frame annotations, sequence level annotations were created based on each participant’s self-report and the observer’s measures. As a result, this dataset is of remarkable interest for those approaches that do not involve any other modality.

A different approach was adopted by the authors of the BioVid Heat Pain Database [11], comprising multi-modal data acquired from sensors (GSR, EMG, ECG, and EDA) and RGB videos of the participant’s face. This database contains four progressively increasing levels of artificially induced pain, along with baseline data, for each of the 87 participants. Although the experiments involved healthy participants, the recorded pain-related expressions were genuine. This database is particularly significant because of the combination of data from at least two different modalities: camera recordings and physiological sensor signals.

More recently, the Multimodal EmoPain Dataset [12] was introduced. This dataset focuses on chronic pain and includes data from 22 individuals with chronic lower back pain and 28 healthy individuals for comparison. These participants engaged in various physical exercises within a realistic rehabilitation environment. The dataset encompasses high-resolution multi-view videos, multi-directional audio streams, three-dimensional motion capture data, and EMG signals from back muscles. The recorded data underwent dual annotation processes. The first set of labels represents continuous pain levels observed by eight annotators, ranging from 0 (minimal pain) to 1 (maximal pain), based on facial expressions. The second set of labels identifies six pain-related body behaviors (guarding or stiffness, hesitation, bracing or support, abrupt action, limping, rubbing or stimulating) as defined by six rehabilitation experts.

Although the primary goal of utilizing deep learning in pain assessment is to determine the level of pain experienced by nonverbal patients, scholarly research also aims to evaluate the patients’ oral expression to establish the pain severity they experience. In [13], a new database was presented presented, consisting of 844 recordings obtained from 80 subjects, referred to as the Dusseldorf Acute Pain Corpus. The objective of their study was to conduct a three-level classification of pain severity (mild/moderate/severe) utilizing support vector machines (SVMs) and long short-term memory recurrent neural networks (LSTM-RNNs) as classifiers. Different features are extracted for audio signal processing and speech recognition, including Mel-Frequency Cepstral Coefficients (MFCCs) and deep spectrum representations obtained by processing the spectrogram of audios using the Convolutional Neural Network VGG16 [14]. The results obtained in this study were satisfactory, concluding that the classification of pain by signal analysis is very positive and will give rise to different studies in the future.

### 2.2. Methods

Automatic pain recognition necessitates at least one sensory input channel to supply the computer with information, often referred to as a modality. These modalities can be broadly categorized into behavior and physiology. Behavioral modalities encompass facial expressions, body movements (such as guarding, rubbing, restlessness, and head movements), vocalizations (like crying or moaning), and spoken words (which can be transcribed by speech recognition and may contain self-reported information). Within the physiology domain, relevant modalities include brain activity, cardiovascular activity, and electro-dermal activity. Additionally, conventional direct human–computer interfaces like keyboards or touch displays can be employed to gather self- or observer-reported pain, potentially along with related activities or contextual information, thus complementing the information gathered from other modalities.

In addressing the aforementioned challenge, one of the prevailing methodologies involves the application of Convolutional Neural Networks (CNNs). This widely adopted approach is extensively discussed in [15], wherein networks like SANET [16] and SDNET [17] are employed. SANET excels at automatically discerning spatial attributes such as color, while SDNET specializes in extracting shape-related features, like facial contours. These networks play a pivotal role in both pre-processing the input images and extracting crucial features. Subsequently, a learning phase is initiated to process these extracted features and compute a pain score in accordance with the Prkachin and Solomon Pain Intensity (PSPI) scale. This comprehensive process forms the backbone of our methodology for addressing the identified issue.

In [18], the authors present a study with extensive data demonstrating that pain measurement scales are generally helpful but their reliability is compromised when used for individuals with cognitive disabilities. Avoiding subjective evaluations and utilizing clear, value-neutral language, the authors provide a logically structured and concise explanation of their findings. Furthermore, they adhere to conventional academic structure and formatting while also employing precise subject-specific vocabulary. Overall, their study serves as a valuable contribution to the field of pain measurement. It is recommended that pain assessment be multi-modal, incorporating the examination of variables such as body posture, facial expressions, and psychological parameters, as these factors can influence the representation of pain. This project utilizes various techniques to extract characteristics from the analyzed data, including audio, video, and trapezius Electrocardiograms, which are subsequently fused in different phases for classification.

A system that processes information from a single modality is known as a unimodal system; when it utilizes multiple modalities, it is referred to as a multimodal system. A promising approach involves combining modalities within a multimodal system. Diverse information sources can complement each other, potentially leading to enhanced specificity and sensitivity. In general, if the individual modalities demonstrate sufficiently strong predictive performance, their fusion tends to yield improved results. This has been demonstrated in various studies, including the combination of facial expression and head pose [19,20]; EDA, ECG, and sEMG [20,21]; video, EDA, ECG, and sEMG [20,22]; video, RSP, ECG, and remote PPG [23]; video and audio [24]; and MoCap and sEMG [25].

## 3. Data Analysis from Acquisition Sensors

For the experiments in the subsequent sections, we selected the previously introduced BioVid Heat Pain Database [11]. This section provides in-depth explanations and analyses of the selected databases.

In the upcoming section, we delve into the examination of data obtained from sensors from the BioVid Heat Pain Database [11]. The following subsections aim to offer comprehensive insights and detailed analyses of the chosen database. Notably, the UNBC-McMaster Shoulder Pain Expression Archive Database [10] was not employed in these analyses due to its lack of sensor data.

### 3.1. Data Preparation

The BioVid dataset contains videos of 90 healthy adults between the ages of 20 and 65. The dataset was created by the Neuroinformatics Technology Group at the University of Magdeburg and the Medical Psychology Group at the University of Ulm [11,26]. Participants underwent controlled experiments in which thermal stimuli were applied to various body regions, including the forearm and leg. The database is remarkably diverse and includes extensive participant information, including age, gender, medical history, and pain sensitivity. In addition, subjective pain responses were documented, including ratings of pain intensity and discomfort. Objective data including ECG, EMG, skin conductance and heart rate were collected.

The database is organized into five subsets (A, B, C, D, and E), each containing some different data. Some of them include facial EMG, differences in the muscles receiving the thermal stimulus, or even the emotional reactions of the patients induced by watching videos to analyze reactions to situations of sadness, anger, fear, happiness or a neutral state. The BioVid database is considered one of the largest in the field of pain, making it a viable option for experimentation. It offers a wealth of information on subjective pain from patients and data from sensors and measurements from experts.

Subsets A and B of the BioVid dataset were analyzed to explore variables and look for correlations. This provides a robust basis for examining responses to pain stimuli and investigating the relationship between biomedical variables and facial expressions in painful situations. The patient data are structured as follows:

- Part A: Pain Stimulation without Facial EMG (short time windows): It includes frontal video and contains biomedical signals in the form of raw and pre-processed data, such as GSR (Galvanic Skin Response), ECG (Electrocardiogram) and EMG (Electromyography) in the trapezius muscle. It consists of a total of 8700 samples from 87 subjects. The data are divided into 5 classes with 20 samples per class and subject. The time windows have a duration of 5.5 s.
- Part B: Facial EMG Pain Stimulation (partially masked face, short time windows): This part comprises 8600 samples and includes frontal video alongside biomedical signals in the form of both raw and pre-processed data, including GSR, ECG, and EMG. Notably, these signals are collected from additional muscles, such as the corrugator and zygomatic muscle. The dataset involves 86 subjects, with 84 of them also present in Part A. Similar to Part A, it is segregated into 5 classes, each consisting of 20 samples per class for every subject.

The merging of the two aforementioned subsets resulted in a larger database for analysis. In this process, the time series of each of the samples was examined in detail, and key statistics were calculated, including the mean, minimum, maximum and standard deviation of these time series. In total, 3480 samples were processed and combined, allowing a more comprehensive assessment of subjects’ responses to pain stimulation and the relationships between various biomedical variables and facial expressions in painful situations. To better understand the significance of the biomedical variables used in this study, we provide the following:

- GSR (Galvanic Skin Response) measures the electrical conductivity of the skin, which can change in response to emotional or physiological stimuli. An increase in GSR may suggest an increase in emotional or physiological activity.
- ECG (Electrocardiogram) records the electrical activity of the heart, providing information about heart rate, heart rhythm and the electrical activity of the heart muscle. The emotional state can also affect it.
- Trapezius EMG (Electromyography of the trapezius muscle) measures the electrical activity in the trapezius muscle. The activity may escalate proportionately with muscle tension or strain. It is utilized in this context to evaluate the muscle response of participants to pain stimulation, which can imply the strength of the physical reaction to pain.

### 3.2. Variable Analysis

The time series of these three variables was thoroughly analyzed to identify patterns using both additive and multiplicative seasonality analysis techniques for each variable.

**Figure 1:**
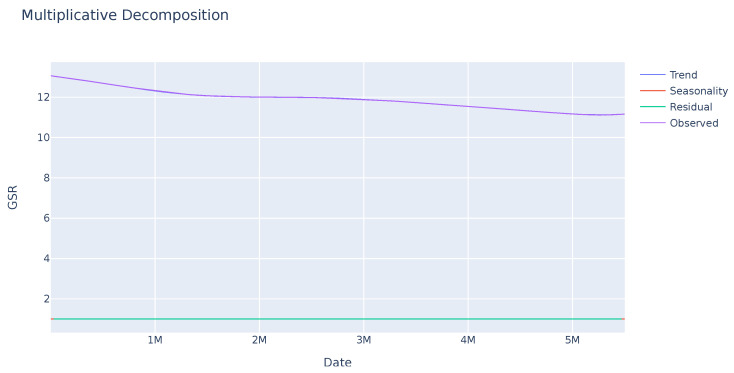
Seasonal trend of the GSR variable of a sample. The observed value remains stable. The seasonality and residual values, in red and green successively, indicate that there is no pattern or significant deviations in this variable.

**Figure 2:**
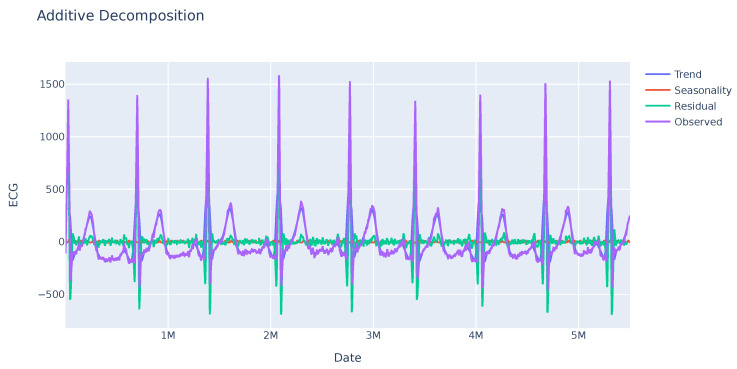
Seasonal trend of the ECG variable of a sample. This variable shows a clear trend sustained over time, in purple color. In addition, high residual values appear in some regions of the series.

A seasonality analysis was performed on the GSR variable, analyzing the observed data, trend, and seasonality. The results indicate an absence of seasonality or a clear trend as seen in Figure 1. Further investigation is needed to identify other potential patterns in specific sections of the sequence. In contrast, the ECG variable exhibits greater variability with a clear seasonality trend as shown in Figure 2. This enables analysis of the deviation from anticipated data values and their subsequent use in evaluating user disruptions.

**Figure 3:**
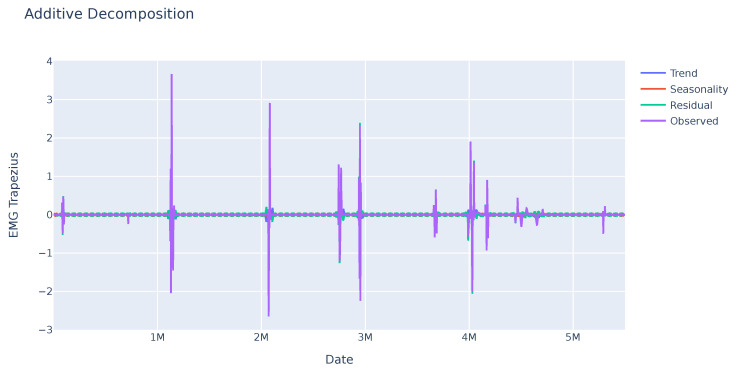
Seasonal trend of the EMG variable of a sample. No pattern is identified in this variable, but some abrupt changes stand out at some points in time that may be significant.

As it can be seen in Figure 3, the EMG data do not show any seasonality, but there are large changes in the values at certain times, which can be used to detect changes in muscle tension and may be related to the feelings the person is experiencing.

The distributions of the mean, maximum, minimum and standard deviation values for all samples were then analyzed on a variable-by-variable basis. The violin plot is another way of displaying the same distribution, which allows us to visually evaluate the two distributions.

**Figure 4:**
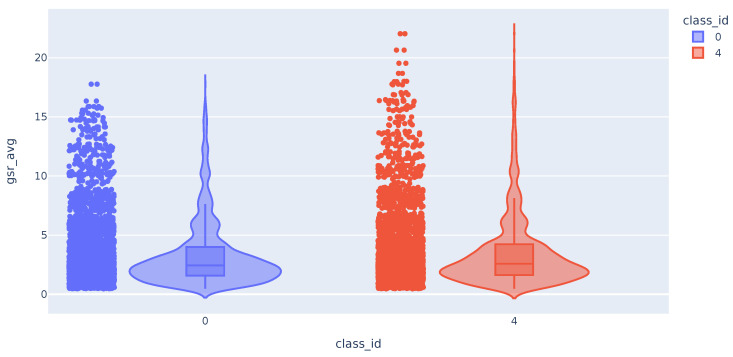
Violin plot with the distribution of GSR variable.

The distribution of the mean values of the GSR time series appears to be the same for the samples of class 0 (no pain) and class 4 (maximum pain). As can be seen in Figure 4, although the tail of the class 4 distribution is longer, they have a similar shape. The distribution of the maximum and minimum values of the GSR variable also does not differ between the different classes. On the other hand, the distribution of the standard deviations of the GSR differs between the classes. We can see that high std values for this variable correspond exclusively to samples in class 4 (maximum measured pain).

**Figure 5:**
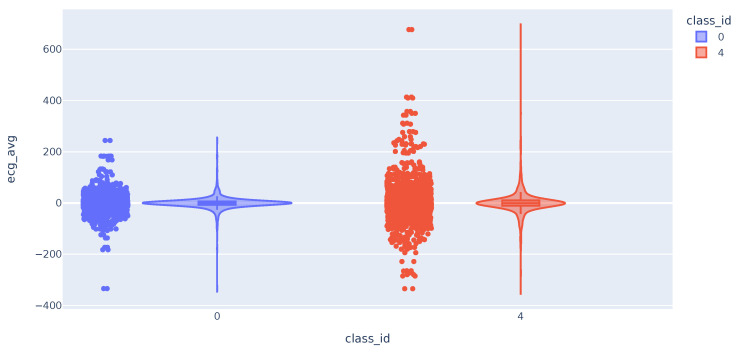
Violin plot with the distribution of ECG variable.

We then analyze the distribution of the Electrocardiogram (ECG) time series metrics. The distribution of the mean values follows a broader bell curve for class 4 samples. In some cases, the maximum and minimum values of the class 4 samples are higher and lower, respectively. This difference in values from the mean may help to distinguish cases of users with high pain. As expected, the highest deviation values are almost all for class 4 samples (Figure 5).

**Figure 6:**
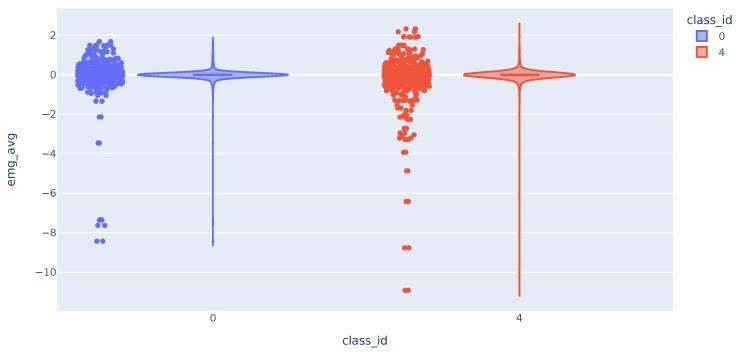
Violin plot with the distribution of EMG variable.

The distribution of the trapezius EMG variable is similar in both classes as shown in Figure 6. The three variables have comparable distributions when comparing data from classes 0 and 4. The data shows that values deviating the most from the mean are mostly from class 4. This suggests that these variables can potentially differentiate between pain presence or absence. The challenge arises from the large cluster of data around similar values, which results in significant overlap in distribution curves across various classes. As a result, accurately classifying most of the samples becomes challenging.

**Figure 7:**
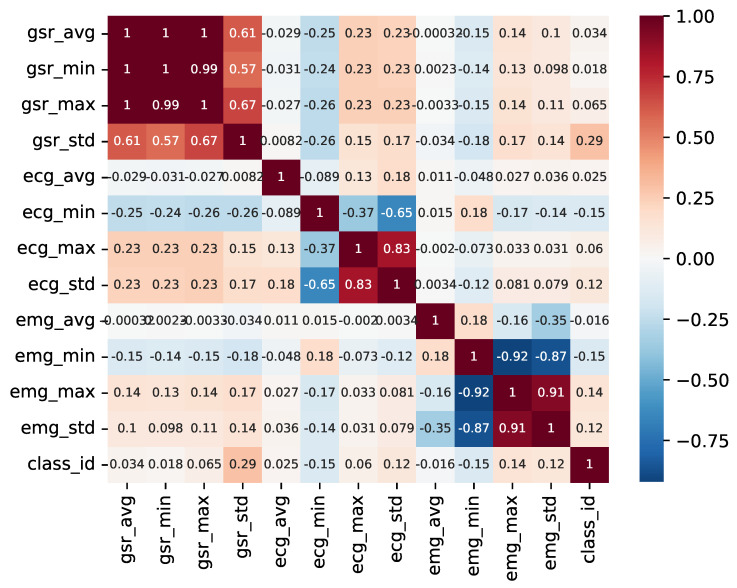
Pairwise variable heatmap.

To tackle this, a pairwise correlation analysis was conducted to identify the variables that display the highest correlation with the classification and to check whether certain variables provide redundant information. Figure 7 illustrates the outcome of this analysis.

As observed, there is a minimal correlation between individual variables and the sample class. Furthermore, it is evident that various metrics (mean, maximum, and minimum) derived from the time series of the GSR variable exhibit direct correlations, with the distribution of each being a linear combination of the others. In contrast, the metrics derived from the ECG variable display greater variability and may hold more relevance in the detection of anomalies and fluctuations within the time series.

### 3.3. Machine Learning Methods

**Figure 8:**
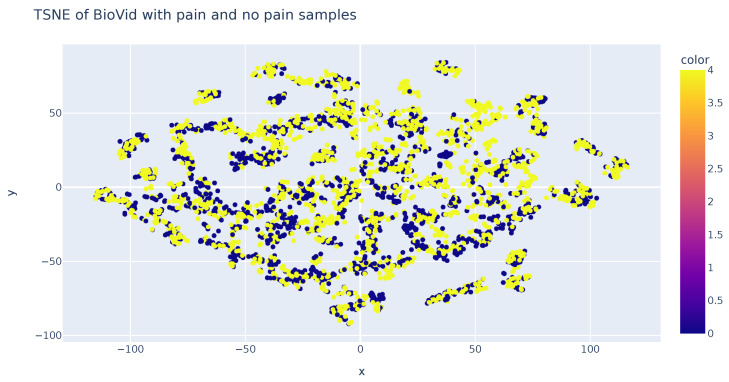
TSN-E representation of features in each class.

An analysis was performed using the T-SNE algorithm to reduce the data’s dimensionality and enable visual representation. This method allows for the representation of samples in both two and three dimensions. The accompanying figure demonstrates sample representation using two T-SNE components. It becomes evident that the samples are consistently distributed in the two-dimensional space, a feature that can also be observed in the three-dimensional representation. However, it should be noted that this technique does not aid in the classification of samples based on their respective classes. Consequently, this analysis concludes that dimensionality reduction algorithms, specifically T-SNE, fail to sufficiently distinguish variations in sample values among each class (Figure 8).

In conclusion, conventional machine learning models were implemented for the purpose of data classification. Initially, we utilized logistic regression, and then examined a variety of algorithms, including newton-cg, lbfgs, liblinear, sag, and saga. Our experiments continued with support vector machines (SVMs), utilizing four distinct kernels: linear, polynomial, rbf, and sigmoid. As our final approach, we explored decision trees by varying the maximum branching depth ranging from 3 to 12.

The summarized results can be found in the appended Table 1, which presents the accuracy of each model during the testing phase. Notably, decision trees exhibited the highest performance, achieving an accuracy of up to 79.45% with a maximum depth of 12 branches. These findings underscore the relevance of the dataset’s provided information for pain detection in individuals. Nevertheless, they also imply that to further enhance accuracy, it may be imperative to incorporate supplementary data and explore more intricate models in future research endeavors. Video data could be used with deep learning methods to improve these results.

## 4. Per-Frame Analysis

As the initial task in the experimentation within this study, we tackled the challenge of pain estimation from static images or frames, involving the assessment of pain levels based on subjects’ facial expressions using state-of-the-art computer vision techniques. This section is structured as follows: Section 4.1 describes the data preparation followed for the experiments. Section 4.2 describes the proposed models for experimentation. And finally, Section 4.3 presents and discusses the results.

### 4.1. Data Preparation

**Figure 9:**
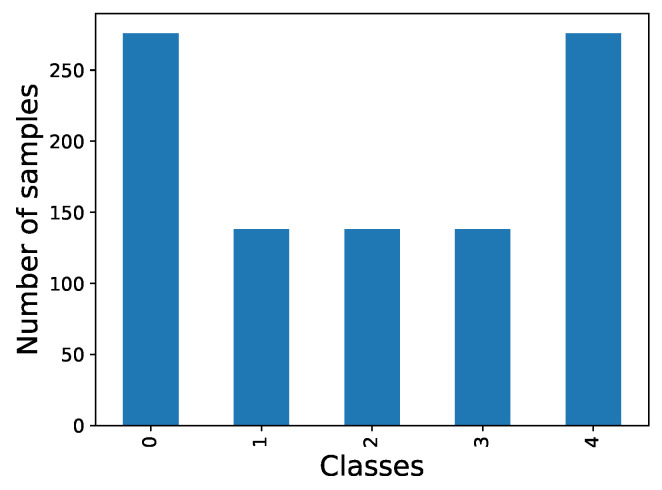
Distribution of frames per class on Biovid dataset.

The first step in the process entails analyzing the two aforementioned datasets and preparing the data for per-frame analysis. In the case of the BioVid dataset, which solely contains videos with video-level annotations, a subset was selected considering different classes, genres and ages as represented in Figure 9. From this subset, frames were extracted and uniformly classified into the same category for analysis, corresponding to the video class.

**Figure 10:**
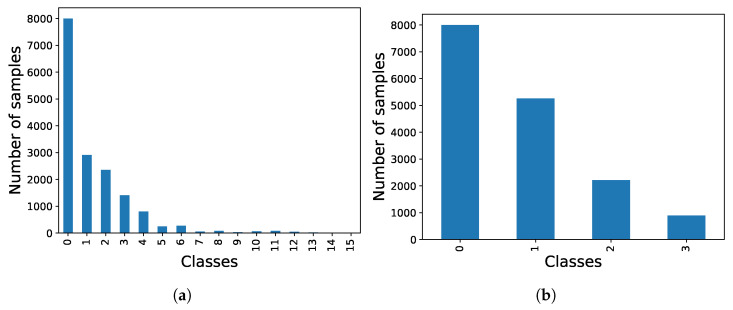
Comparison of UNBC-McMaster dataset class distributions. (**a**) Initial distribution (class 0 limited to 8000 samples for visualization). (**b**) Balanced distribution.

In contrast to the previous dataset, the UNBC-McMaster dataset provides annotations per frame, which is expected to result in improved image classification. However, the primary drawback lies in the large number of classes that need to be classified and the imbalanced distribution among these classes as illustrated in Figure 10a. To mitigate issues related to overfitting or class bias, the PSPI values were organized as depicted in Figure 10b, clustering the different labels into four main groups. Additionally, the number of samples in class 0 (baseline) was capped at 8000 to achieve a balanced dataset.

### 4.2. Proposed Models

After the selection of datasets for experimentation, a fine-tuning process was initiated. This involved testing various architectures, as elaborated upon below, categorized into two primary groups: Convolutional Neural Networks (CNNs) and Transformer-type architectures. The proposed CNN models are presented in Section 4.2.1 and Transformer-based models are presented in Section 4.2.2.

#### 4.2.1. Convolutional Neural Networks (CNNs)

Convolutional Neural Networks are widely utilized architectures in the computer vision field for extracting information and features from images and learning patterns within them.

We selected models that vary significantly in both the number of parameters and depth. This approach will allow us to examine how these variations between the models impact the classification.

- VGG16 [14]. This neural network comprises 16 layers, including 13 convolutional layers for feature extraction and 3 fully connected layers for classification. It utilizes 3 × 3 filters and is composed of a total of 138 million parameters.
- MobileNetv2 [27]. The architecture of this network is based on residual blocks, where the input of a layer is integrated into the output, enabling it to learn the disparities. This block type facilitates the reduction of computational complexity and the number of parameters, rendering this architecture a highly viable choice for pain estimation, given its suitability for deployment on various mobile devices. However, this decrease in computational load may impact the results, resulting in lower accuracy compared to other approaches.
- ResNet50V2 [28]. The primary feature is its capability to train extremely deep networks without compromising performance. It is extensively employed in feature extraction and image classification. Due to its depth, it demands a substantial amount of computational power.
- InceptionV3 [29]. This network is distinguished by its use of Inception modules. These modules operate as multiple filters simultaneously applied to the input. The outcomes of these filters are concatenated, forming the module’s output. While this enhances accuracy, it also escalates the parameter count and computational demands. The characteristics of this model, particularly its proficiency in extracting both global and local features, make it a powerful model, heavier than others such as MobileNetV2 (InceptionV3 has 23.9 million parameters) but balanced and widely used in image classification.
- Xception [30]. The name of this model derives from the concept of ‘separable convolutions’. It is a variation of the InceptionV3 model (with 22.9 million parameters, very close to the 23.9 million of InceptionV3) that substitutes the Inception modules with separable convolutions. This approach involves conducting convolutions in two steps: depth convolution and point convolution. This technique is employed to alleviate the computational load without significantly compromising performance.

#### 4.2.2. Transformers-Based Architectures

Unlike the traditional convolutional architectures described above, these models are based on Transformers [31], originally designed for Natural Language Processing (NLP). Transformer-based models currently represent the state of the art in NLP tasks, replacing the previous models, which is why they have started to be used for other modalities such as vision.

- Vision Transformer (ViT) [32]: This model divides the image into small patches that are treated as sequences of tokens, the type of input used by the Transformer layers. In this way, the model can learn complex relationships between the different patches and understand the structure of the image as a whole. This ability also allows it to learn abstract and contextual patterns on unlabeled data (self-supervised learning), so we can pre-train on large datasets and then perform fine-tuning on a specific task, such as pain estimation in this case.
- Bidirectional Encoder representation from Image Transformers (BEiT) [33]: The authors introduce a masked image modeling task for the pretraining of vision Transformers. This model partitions the image into smaller patches, with some of these patches deliberately masked. This deliberate masking introduces corruption into the image, requiring the model to subsequently reconstruct the original image from the corrupted version. Following this pretraining phase, the model becomes suitable for deployment in downstream tasks, which can be pain estimation in this case.
- Swin V2 [34]: This is the largest dense vision model, boasting 3 billion parameters. The authors present three primary techniques aimed at enhancing the results: a residual-post-norm approach combined with cosine attention to enhance training stability, a log-spaced continuous position bias method designed to effectively facilitate the transfer of pre-trained models from low-resolution images to downstream tasks with high-resolution inputs, and a self-supervised pretraining method, known as SimMIM, to reduce the requirement for extensive labeled images.

### 4.3. Results

After completing the work on the datasets and selecting the models to be tested, the final step involves training the models, obtaining the metric results, and analyzing them to draw conclusions.

The first experimentation was performed using the Biovid dataset; this attempt did not yield the expected results. The accuracy of CNN-based models did not surpass 20% when distinguishing between the detection of specific classes (PA1, PA2, PA3, or PA4) and non-detection (class BL1). In fact, even in this binary classification scenario, the accuracy did not exceed 50%. In essence, these outcomes are no better than random chance, leading us to the conclusion that the model did not learn anything meaningful.

In contrast, experiments with Transformer-based architecture produced promising results. When we mixed participants in the dataset, we achieved 97.04% accuracy on the test set and 98.36% accuracy on the validation set. However, when training on one participant and evaluating on another, we reached 100% accuracy on the validation set but only 20% on the test set. This discrepancy suggests overfitting to the training data and implies that this dataset may not be suitable for frame-based classification.

On the other hand, in the case of the UNBC-McMaster dataset, the results were favorable. This outcome was expected, as this dataset was particularly well suited for the task from the outset, given that the data are in image form rather than video. As mentioned previously, this dataset will be tested using two subsets: one with full labeling and another that clusters different labels while reducing the samples of the most common ones. This approach results in a more balanced dataset with fewer labels, making it easier for the models to learn how to estimate pain.

Regarding the results over the UNBC-McMaster dataset test set presented in Table 2, as evident from the table and as anticipated in the experimental design, the best results were achieved using the clustered version of the dataset. It is worth noting that Transformer-based architectures outperformed classical Convolutional Neural Networks in this task, even in cases involving the full dataset with 16 labels. The BEiT model delivered the best performance, achieving over 96% accuracy on the test set, slightly outperforming the ViT model.

Taking these results into consideration, we gathered more detailed information regarding the metrics corresponding to the optimal model, specifically the BEiT model. It demonstrated a notable 96.37% accuracy on the test set. The additional significant metrics encompass a 94.61% F1 Score, 94.67% precision, and 94.55% recall. These metrics exhibit slightly inferior performance compared to the accuracy metric.

## 5. Video Analysis

After analyzing individual frames, we proceeded to analyze the complete video sequences from part A of the BioVid database. The aim was to assess whether analyzing temporal sequences that provide a broader context of the events can enhance the previous results. To achieve this, we conducted experiments using three video Transformer models, as they currently outperform other models for video classification, such as convolutional networks (CNNs) or recurrent networks (RNNs), in terms of accuracy. This section is structured as follows: Section 5.1 describes the data used for the experiments. Section 5.2 describes the proposed models for experimentation. And finally, Section 5.3 presents and discusses the results.

### 5.1. Data Analysis

**Figure 11:**
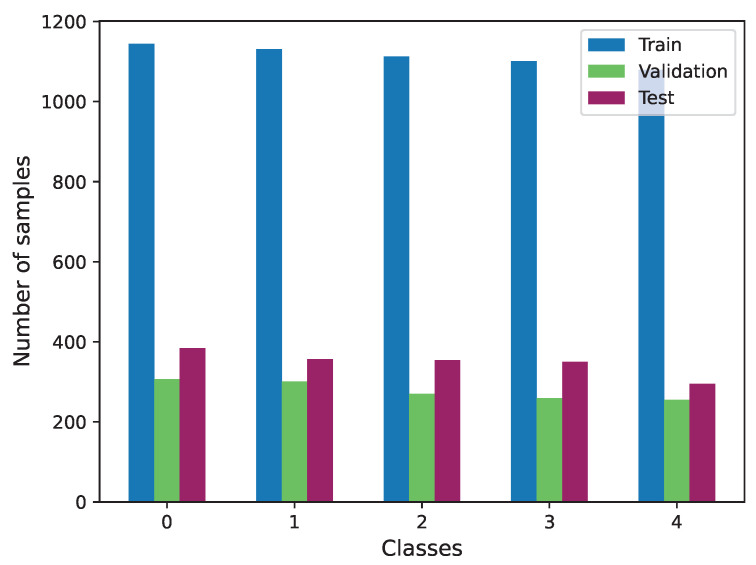
Class distribution of Biovid train, validation and test subsets.

For this task, we exclusively utilized the Biovid dataset, as it is the sole source of video information. In this case, we specifically used part A of this dataset. Given the balanced distribution of data across labels within this part, no additional data preparation was required. The distribution of the three subsets used is illustrated in Figure 11.

### 5.2. Proposed Models

In this context, we required models with the capacity to process visual temporal information, specifically sequences of frames or images. The models previously mentioned are unsuitable for this task, as they are designed exclusively for single-frame processing. Therefore, we chose to work with Transformer-based architectures, which have demonstrated exceptional performance in other applications. In this case, we applied them to video processing, referred to as Video Transformers. Video Transformers distinguish themselves from other architectures due to their unique ability to capture long-term temporal relationships within sequences. This subsection provides a concise overview of three state-of-the-art models that will be employed in our experimental work.

#### 5.2.1. Video Masked AutoEncoder (VideoMAE)

Video Masked AutoEncoder (VideoMAE) [35] architecture, although not a Transformer itself, collaborates with them to deliver state-of-the-art results. The authors investigated the utilization of autoencoders for the self-supervised pre-training task necessary for Transformers. To extract features, this model employs the vision Transformer introduced in the previous section, namely ViT.

#### 5.2.2. TimeSformer

The TimeSformer [36] is another architecture based in the Transformer architecture, purposefully designed to handle temporal sequences. This model extends the principles of the ViT (Vision Transformer) into the temporal domain for application in video processing. Because it is exclusively built on self-attention layers, there is no need for convolutional layers. Notably, the authors conducted a comparative analysis of various attention mechanisms within this model. Among these, the ‘divided attention’ approach stands out, where network blocks employ separate temporal and spatial attention, resulting in the most favorable outcomes.

#### 5.2.3. ViViT

The ViViT model [37] was introduced in response to the success of image Transformers. The authors proposed a range of Transformer-based models for video classification:

- Transformer Encoder. This is an extension of ViT into the temporal dimension.
- Factorized Encoder. In contrast to the previous model, this approach does not use the same encoder for all videos. Instead, each video is divided into several chunks. Spatial attention is initially applied to each clip, and the resulting vectors serve as input to a second encoder, this time with a temporal focus. Each clip is assigned an index for proper identification.
- Factorized Self-Attention. This is similar to the first model but with a distinction in the attention computation, which occurs in two phases: spatial attention followed by temporal attention.
- Factorized Dot Product. This is another variation of the first model, with modifications at a lower level. Specifically, within the attention layers, scalar product operations are divided, with half performed on spatial tokens and the remaining half on temporal tokens.

Among these architectures, the second one, Factorized Encoder, yields the most promising results.

### 5.3. Results

After the selection of the different models, our next undertaking is to initiate the training process for these models, with the intention of subsequently testing them using the test subset from the Biovid dataset. The outcomes of this testing are presented in Table 3.

After conducting the experiments, we can conclude that, within the utilized dataset, analyzing the context of the entire video does not yield the anticipated results. This can be attributed to the fact that the videos used only capture the patient’s face, revealing signs of pain during very specific moments (frames).

The application of these techniques may prove highly beneficial when working with other types of videos that offer more contextual information, such as preceding actions that trigger the patient’s specific reactions.

## 6. Discussion

Pain detection and assessment play a pivotal role in healthcare, aiding clinicians in understanding and addressing patients’ physical discomfort and emotional distress. Traditional methods of pain evaluation often rely on subjective self-reporting, which can be challenging when dealing with individuals unable to communicate effectively or accurately express their pain levels. The results presented in this study highlight the ability to use facial expressions to assess pain using different data sources and models, such as Transformer-like architectures. We show how these methods hold promise for distinguishing different levels of pain. However, our exploration also sheds light on the crucial limitations and factors that influence the relevance and efficiency of these approaches.

### 6.1. Temporal Context and Transformer-Style Architectures

While our work showcases the potential of leveraging facial expressions for pain detection using Transformer-style architectures, the limitations surrounding the temporal context within the BioVid database raise important considerations. The incorporation of temporal information did not yield a substantial improvement in pain detection accuracy. This indicates the complexity of capturing nuanced changes in pain expressions over time, which remains a challenge for current video classification models. Further exploration is needed to refine temporal modeling techniques that better capture the dynamic nature of pain expressions.

### 6.2. Context Impact

One notable insight is the observation that this approach may find substantial utility in scenarios where the source of pain is visually evident in the video. This implies that the efficacy of the proposed method may be context-dependent, with its strength being more pronounced when the pain-inducing event is visually conspicuous.

In light of these observations, we propose the exploration of multi-modal approaches for enhanced pain detection accuracy. Combining facial expressions with physiological data obtained through sensors capable of measuring direct physiological impulses could provide a more comprehensive understanding of the patient’s pain experience. This integration of different modalities may contribute to a more robust and reliable pain detection system.

### 6.3. Proposed Future Directions and Research Opportunities

Building on our findings, we propose exploring multi-modal approaches that combine facial expressions with physiological data obtained through sensors capable of measuring direct physiological impulses. Integrating multiple modalities, such as heart rate variability, skin conductance, or brain activity, alongside facial expression analysis, could offer a more comprehensive understanding of an individual’s pain experience. This integration holds potential for creating a more robust and reliable pain detection system, surpassing the limitations of visual cues alone.

## 7. Conclusions

In conclusion, our extensive exploration of pain assessment methods yielded encouraging results. By merging data from biomedical sensors and state-of-the-art computer vision techniques, our study established a new benchmark in the field. The thorough analysis of both the UNBC-McMaster and BioVid datasets provided a solid foundation for future research with an accuracy of over 30% using video prediction models and over 93% for the 16 classes of the UNBC-McMaster dataset.

Our examination of the sensor-derived data indicates that the identifiable features within the selected signals provide sufficient insight to assess increased pain levels in patients. However, additional data may be required for the accurate detection of lower pain thresholds. Regarding the analysis of facial expressions in the current framework, it appears that these features can be exploited by Transformer-like architectures to measure the level of pain. However, the accuracy of the video classification models suggests that the temporal context within the BioVid database may not significantly improve the results. Nonetheless, this methodology could prove to be significantly influential when applied to videos that explicitly depict the source of pain (e.g., a person falling to the ground).

In addition, we advocate the adoption of multimodal approaches that fuse a patient’s facial expressions with data from sensors capable of directly measuring physiological impulses. Alternatively, the use of algorithms that detect variations in video inputs, such as optical flow, has shown remarkable effectiveness in other video classification tasks and could serve as a valuable avenue for exploration. However, the subjective nature of pain perception demands a comprehensive understanding of cultural, psychological, and contextual factors.

## Figures and Tables

**Table 1:** Results of the tested machine learning methods using the BioVid dataset.

Model	Params	Classes	Precision (Test)
Logistic Regression	newton-cg function	0, 4	72.91%
Logistic Regression	liblinear function	0, 4	76.06%
SVM	kernel linear	0, 4	71.34%
Decision tree	max_depth = 9	0, 4	76.65%
Decision tree	max_depth = 12	0, 4	79.45%

**Table 2:** Results for the various models on the UNBC-McMaster test set.

Model	Classes	Accuracy	Loss
VGG16	Clustered, 4	94.75%	0.131
MobileNetV2	Clustered, 4	87.61%	0.522
ResNet50V2	Clustered, 4	93.88%	0.197
InceptionV3	Clustered, 4	92.87%	0.206
Xception	Clustered, 4	80.75%	0.757
ViT	Clustered, 4	96.37%	0.110
	Full, 16	91.72%	0.260
**BEiT**	**Clustered, 4**	**96.82%**	0.084
	**Full, 16**	**93.86%**	0.166
Swin V2	Clustered, 4	93.77%	0.157
	Full, 16	91.39%	0.255

**Table 3:** Results for the various models on the Biovid test set.

Model	Accuracy	Loss	Training Loss
TimeSformer	23.05%	1.60	1.64
VideoMAE	25.06%	1.58	1.56
**ViViT**	**30.07%**	1.49	1.49

## Data Availability

The datasets used during this study are available by contacting the authors of the datasets and signing agreements.
